# Adherence and retention to the self-managed community-based Step Into Health program in Qatar (2012–2019)

**DOI:** 10.3389/fpubh.2022.927386

**Published:** 2022-09-15

**Authors:** Bryna C. R. Chrismas, Lina Majed, Abdulla Saeed Al-Mohannadi, Suzan Sayegh

**Affiliations:** ^1^Department of Physical Education, College of Education, Qatar University, Doha, Qatar; ^2^World Innovation Summit for Health, Qatar Foundation, Doha, Qatar; ^3^Aspetar Orthopedic and Sports Medicine Hospital, Doha, Qatar

**Keywords:** physical activity, public health, wearable technology, pedometer, smartphone application, walking

## Abstract

**Purpose:**

Investigate adherence and retention to the “Step Into Health (SIH)” initiative (www.stepintohealth.qa [website access only available from within the State of Qatar]), a Qatari self-managed community-based health program, from 2012 to 2019.

**Methods:**

Participants (16,711; 16–80 years; 37% females, 34% Qatari) used a pedometer or smartphone application (app) to measure step count. Absolute adherence (ADH) and retention (RET) were calculated, with ADH (%) the ratio between number of days data and SIH enrollment length (RET). Linear Mixed Models identified differences in ADH between RET groups, main effects (i.e., sex, device, age, BMI, nationality) and interaction effects for ADH (RET entered as a covariate).

**Results:**

Average ADH and RET to SIH (irrespective of sex, age, device and BMI) was 50% (±31%), and 16% (±20%), respectively, with ADH differing significantly between RET groups (*F* = 460.2, *p* < 0.001). RET (as a covariate) revealed a significant main effect for device (*F* = 12.00, *p* < 0.001) and age (*F* = 4.31, *p* = 0.001) on ADH observed. There was a significant association between RET and sex (*p* < 0.001), device (*p* < 0.001), and age groups 16–25 y (*p* < 0.001), and 26–35 y (*p* < 0.001). There were no significant main effects for sex or BMI on ADH, and no interaction effects (*p* ≥ 0.21) observed.

**Conclusions:**

Follow-up data (e.g., interviews, focus groups, etc.) determining why differences in ADH and RET are observed appears prudent. To convert those that lapsed and/or abandoned SIH/PA into committed long-term PA adherers. This would be a first step to develop targeted public health promotions and initiatives to enhance health outcomes at a population level.

## Introduction

Public health promotion is a key pillar of the Qatar National Health strategy (1) and Qatar National Vision 2030 (2). Given that 83% of the Qatari population participate in low (<10 METh/wk) or no weekly physical activity (PA) (3), and 41% engage in no activity during the week (3), community-based initiatives to increase participation are vital. Walking is a culturally appropriate activity for Qatar that can be performed in Islamic traditional clothing (i.e., abaya, thobe), and given the climate (i.e., hot, humid, desert), walking can easily be performed across communities, workplaces, and in air-conditioned facilities (e.g., shopping malls), and weather and/or choice permitting, within recreational parks. Breaking up sitting time with moderate intensity walking improved some aspects of cognitive function (4), and cardiometabolic outcomes (5) in Qatari females. This is important, given that 44% of Qatari females achieve ≤ 5,000 steps per day (6). However, limited studies have objectively (i.e., using accelerometers) measured PA in this population (6–9), with longitudinal data (1 year) showing an average of 7,737 ± 5,336 steps/day across adults in Qatar, with significantly lower daily steps in Arab participants (7). These studies obtained data from the community-based Step Into Health (SIH) program, launched in 2012, and designed to increase PA in adults across the state of Qatar. More recently, a SIH smartphone application (app) was developed, and participants were able to choose whether to use the pedometer or the app. The Qatar smartphone market was valued at USD 1.85 billion in 2017, and is likely to reach 7.15 million units by 2025 (10). Therefore, community-based public health initiatives (i.e., SIH) employing a smartphone app, could be purposeful and successful in increasing PA within the Qatari population.

Although wearable technology is attractive due to its objectivity, it is limited, if not worn and/or used correctly, and by the accuracy of the recorded information (11). Increased adherence [(ADH) i.e., wear time and use)] is associated with reduced missing data (11). ADH (or “compliance”) has multiple definitions within the literature (12), most simply, it is wearing/using the device as directed. Additionally, retention (RET) to self-managed PA, is a persistent issue reported within the literature and high attrition rates (25–50%) across numerous well-designed PA studies have been reported (13–15). Lapsing and/or abandonment of PA tracking may occur due to multitude of reasons (16) for example younger age, time, motivation, access to facilities (17–20). Understanding these factors and behaviors is essential, in order to develop a resource-saving adherence-enhancement strategy to increase RET. Furthermore, understanding the barriers and facilitators associated with participation, ADH, and RET to wearable technology, is a first step to develop targeted public health promotions and initiatives to enhance health outcomes at a population level.

Qatar is a wealthy, tribal, honor-orientated and modernized society, with a relatively small, but highly international and diverse population [including Arab's from the Middle East and North Africa (MENA) region] (21). Arab's likely have variable values and core beliefs regarding PA depending on which MENA region they are from. The SIH has already shown that Arabs have lower daily steps compared to non-Arabs (22), but there is currently no data on the differences in ADH and RET. Additionally, community based pedometer studies have primarily been of short duration (<12 weeks), based in and on Western populations (e.g., USA), typically on small clinical sub-populations (23), of white ethnicity (24), and have sometimes utilized self-report data (24). This study is therefore novel given the duration (7 years), multicultural population, and objective data, which would provide unique yet important information for similar MENA countries and other appropriate regions (e.g., South Asia). Furthermore, given the potential inconvenience of wearing the accelerometer (i.e., has to be worn at the hip, removed for washing), compared to the convenience of the smartphone app (which has not been previously investigated), it is important to understand if ADH and RET to the SIH program could have been affected. Therefore, the aim of this study was to understand the factors (e.g., participant characteristics, device used) associated with ADH and RET to the SIH program, as a first step to develop behavior change strategies, and an adherence-enhancement strategy to increase PA within the Qatari population. It is hypothesized that ADH and RET will be greater for males, smartphone app users, non-Arab's, and in the middle to higher age groups.

## Methods

### Participants and study design

Participants were identified from the SIH program (http://stepintohealth.qa; website access only available from within the State of Qatar). The SIH online program (launched 2012 and now closed) was a national self-managed community-based health program, designed to encourage residents of Qatar to walk ≥ 10,000 steps per day. Participants tracked their steps either using a free pedometer (Omron HJ-324U, Omron Healthcare Co., Ltd., Japan), or a smartphone app, and detailed information on the utilization and data upload procedures was clearly provided. The Omron HJ-324U pedometer has been shown to be accurate in measuring step count, irrespective of terrain and walking speed when worn at the waist, chest or arm (25). Pedometer users were advised to wear their pedometer at their waist, and encouraged to upload their data at least once every 20 days (i.e., the maximum memory capacity of the pedometer). The smartphone app was offered as an alternate option to the pedometer as a smartphone pedometer app may improve habitual physical activity levels in older adults (26), and according to meta-analytical data, could have a small yet positive effect on the number of steps (27) compared to traditional pedometers. The app (integrated on Apple and Android) was connected to the health app and motion sensor. Automated reminders (emails and text messages) were sent to participants who failed to upload their data at 14, 21, and 28 days following the last date of uploaded data. Additional health related information (e.g., nutrition, active lifestyle, sleep) was also available *via* the website and mobile application. A customer service phone line for enquiries and support, and dedicated SIH mobile station was available in the Aspire Zone Foundation Sport City. For more detailed information please see the following article (28). Participants' characteristics (i.e., age, height, body mass, nationality) were entered by the individual upon registration.

This longitudinal study included a total of 24,948 participants (16–80 years). Data was extracted from the SIH program from 2012 until 2019 (i.e., up until the point at which COVID-19 pandemic restrictions were implemented). Participants with <30 days of enrollment (1% of the total length of the program) were excluded, therefore, 16,711 participants (37% females; 34% Qatari) were included in the final analyses. A total of 62% of participants utilized the pedometer. Detailed participant characteristics of pedometer vs. smartphone app users are provided in Table 1. Participants were classified into one of six age groups (16–25, 26–35, 36–45, 46–55, 56–65, and ≥ 66 years old). Additionally, body mass index (BMI) was calculated as the ratio between body mass (kg) and height squared (m^2^) and subsequently each participant was categorized as underweight (<18.50 kg·m^−2^), normal weight (18.5 to < 25 kg·m^−2^), overweight (25 to < 30 kg·m^−2^), obese class I (30 to < 35 kg·m^−2^), obese class II (35 to < 40 kg·m^−2^), or obese class III (≥40 kg·m^−2^) according to the international classification system used by the World Health Organization (29). Participants' physical characteristics are provided in Table 2. On average, participants' age was 38 (±11) y and their BMI was 28.03 (±5.32) kg.m^−2^. Participants signed an online informed consent and disclaimer prior to their enrolment. Ethical approval was obtained from Qatar University Institutional Review Board (QU-IRB 1150-EA/19).

**Table 1:** Participant (*n* = 16,711) characteristics of smartphone app users and pedometer users.

**Device**	**Age [(y), median**	**Female**	**Male**	**Arab**	**Non-Arab**
	**(min–max)]**				
Smartphone app *n* = 6,342 (38%)	37 (16–74)	1,716 (10%)	4,626 (28%)	3,347 (20%)	2,995 (18%)
Pedometer *n* = 10,369 (62%)	39 (16–79)	4,481 (27%)	5,888 (35%)	5,797 (35%)	4,572 (27%)

Data is reported as *n* (%) unless otherwise stated.

**Table 2:** Step into health participants' physical characteristics (*n* = 16,711) by age group, retention (RET) group, and sex.

		**Age group (years)**					
	**Sex**	**16–25**	**26–35**	**36–45**	**46–55**	**56–65**	**≥66**
		**(*n* = 1,821; 11%)**	**(*n* = 5,498; 33%)**	**(*n* = 5,435; 33%)**	**(*n* = 2,889; 17%)**	**(*n* = 958; 6%)**	**(*n* = 110; 1%)**
*n* (%)	Male	748 (41%)	3,581 (65%)	3,631 (67%)	1,873 (65%)	606 (63%)	75 (68%) 10,514
	Female	1,073 (59%)	1,917 (35%)	1,804 (33%)	1,016 (35%)	352 (37%)	35 (32%)
Height (m)	Male	1.73 (1.62–1.75)	1.75 (1.67–1.76)	1.73 (0.167–1.75)	1.72 (1.65–1.74)	1.70 (1.66–1.73)	1.70 (1.69–1.70)
	Female	1.62 (1.54–1.63)	1.60 (1.55–1.62)	1.60 (1.55–1.62)	1.60 (1.57–1.61)	1.60 (1.53–1.61)	1.60 (1.56–1.61)
BM (kg)	Male	75 (63–77)	82 (69–85)	80 (71–83)	84 (75–86)	75 (68–76)	75 (68–78)
	Female	64 (59–70)	69 (57–73)	68 (59–72)	66 (60–67)	72 (67–82)	72 (61–75)
BMI (kg·m^−2^)	Male	25 (15–55)	27 (15–54)	28 (15–55)	28 (16–54)	28 (18–44)	28 (20–41)
	Female	25 (15–53)	26 (15–55)	28 (15–55)	29 (18–55)	30 (17–54)	28 (17–39)
		**RET group**					
	**Sex**	**1–10%**	**10.01–20%**	**20.1–30%**	**30.1–40%**	**40.1–50%**	≥**50.1%**
		**(30–293 d)**	**(294–585 d)**	**(586–878 d)**	**(879–1,171 d)**	**(1,172–1,463 d)**	**(1,464–2,928 d)**
*n* (%)	Male	5,709 (54%)	1,770 (17%)	864 (8%)	590 (6%)	427 (4%)	1,154 (11%)
	Female	3,869 (62%)	940 (15%)	416 (7%)	286 (5%)	203 (3%)	483 (8%)

BM, body mass; BMI, body mass index; RET, retention.

Data is reported as median (minimum–maximum).

### Data analyses

Due to the large variation in length of time that the participants were enrolled in the SIH program, custom software in MATLAB (R2015b, MathWorks Inc., US) was used to compute individual values for absolute adherence (ADH) and retention (RET). Additionally, there is a broad range of definitions used within the literature, and no standardized or agreed cut-off points for ADH or RET (30). ADH (%) was calculated as the ratio between the number of days where data was uploaded, and the overall length of enrollment in the SIH program (RET). For each participant, RET represents the time (number of days) elapsed between his/her first and last uploads, relative to the total length of his/her enrolment in the SIH program (total number of days within the 8 years). Participants were classified into RET groups as follows: 1–10% (30–293 d), 10.01–20% (294–585 d), 20.01–30% (586–878 d), 30.01–40% (879–1,171 d), 40.01–50% (1,172–1,463 d), and ≥50.01% (1,464–2,928 d). Table 2 provides detailed information on the number of participants in each RET group.

### Statistical analyses

Statistical analyses were performed using the Statistical Package for the Social Sciences (SPSS) version 26 (IBM, SPSS Inc, Chicago, IL, USA). Prims8 (GraphPad Software, San Diego, CA, USA) was used to create the figures. Normality of all descriptives were evaluated by visual inspection of quantile-quantile (Q-Q) plots (Grafen and Hails, 2002). Linear Mixed Models (LMM) were performed to analyze the differences in ADH between RET groups. Secondly, LMM were used to examine differences in ADH and RET for nationality (i.e., Arab and non-Arab). Thirdly, LMM were used to compare the main effects (i.e., sex, device, age, BMI) and interaction effects for ADH, with RET entered as a covariate. Finally, an ordinal regression model was performed using the generalized linear model. RET was entered as the dependent variable (outcome), and device, age, sex, and BMI were entered as factors (predictors). Family wise error was controlled for using the step down Hommel method. The significance level was set at *p* < 0.05.

## Results

### Adherence

**Figure 1:**
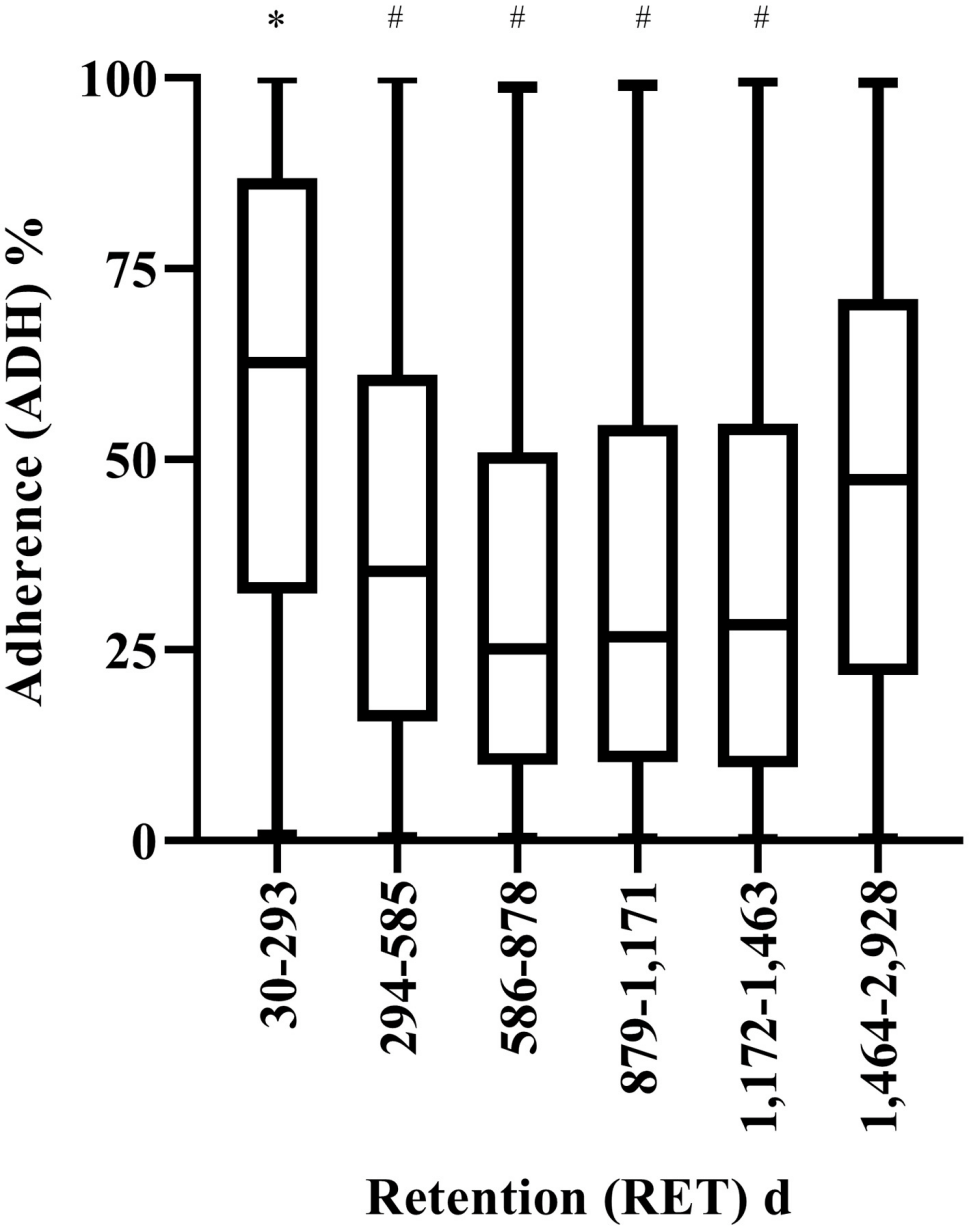
Adherence (ADH) to the step into health (SIH) program for the different retention (RET) groups. *Significantly higher compared to all other groups (*p* < 0.001). # Significantly lower (*p* < 0.001) than the longest RET group (i.e., 1,464–2,928 d). Box and whiskers plot. Line represents median, and whiskers minimum-maximum.

The average ADH to the SIH program (irrespective of sex, age, device and BMI) was 50% (±31%). ADH differed significantly between RET groups (*F* = 460.2, *p* < 0.001) (Figure 1). On average ADH was higher (59%) in the lowest RET group (30–293 d) as compared to all other RET groups: 294–585 d (39%, *p* < 0.001, 95% CI = 18–21%), 586–878 d (32%, *p* < 0.001, 95% CI = 25–28%), 879–1,171 d (34%, *p* < 0.001, 95% CI = 23–27%), 1,172–1,463 d (34%, *p* < 0.001, 95% CI = 23–28%), and 1,464–2,928 d (47%, *p* < 0.001, 95% CI = 11–14%). Additionally, ADH was higher (47%) in the longest RET group (1,464–2,928 d), compared to the those in the 294–585 d (39%, *p* < 0.001, 95% CI = 6–9%), 586–878 d (32%, *p* < 0.001, 95% CI = 13–17%), 879–1,171 d (34%, *p* < 0.001, 95% CI = 10–15%), and 1,172–1,463 d (34%, *p* < 0.001, 95% CI = 10–15%) RET groups. Given that RET affected ADH, RET was entered as a covariate for subsequent analyses.

**Figure 2:**
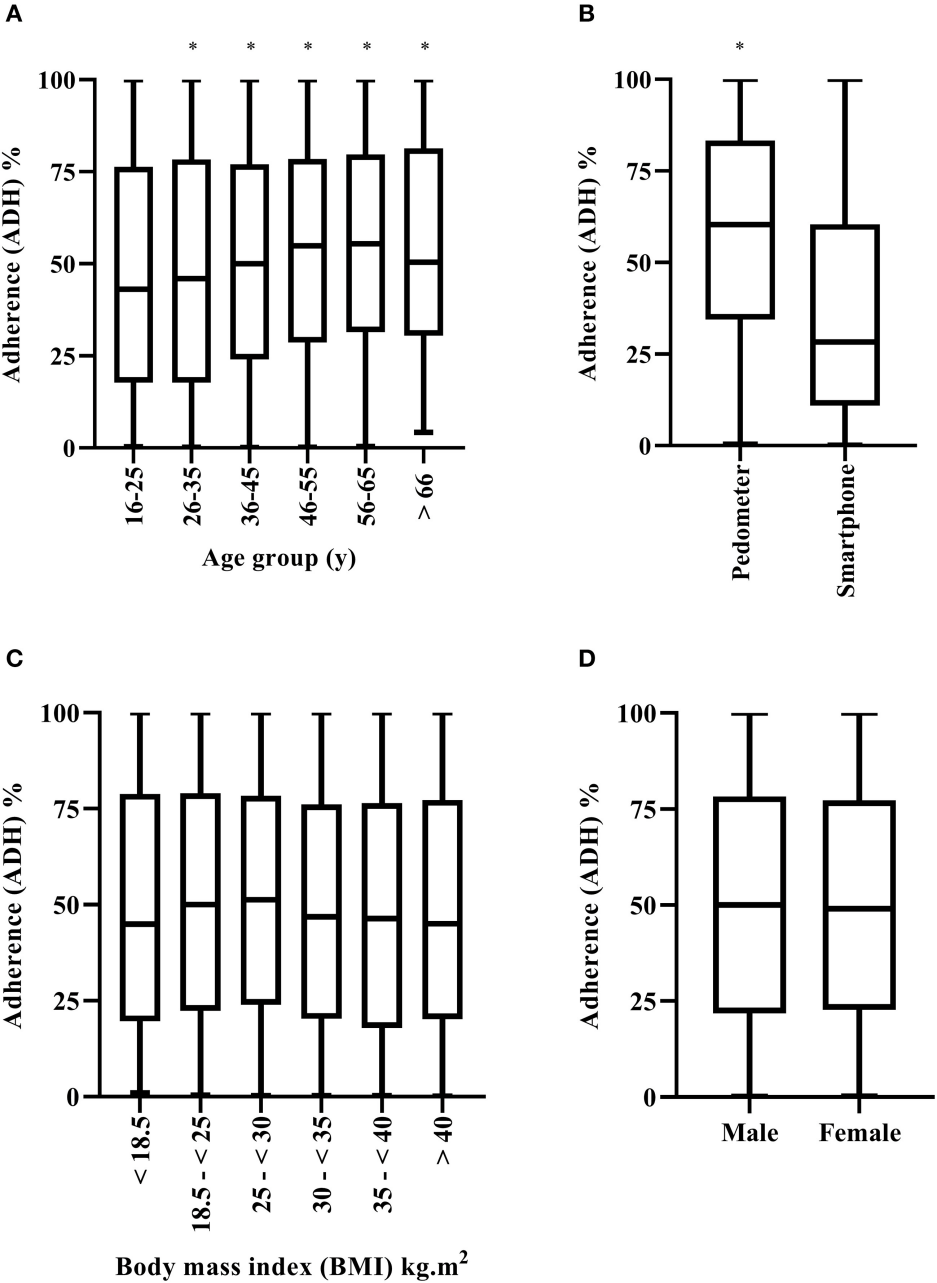
Adherence (ADH) to the step into health (SIH) program by age group **(A)**, device used **(B)**, body mass index [(BMI), **(C)**] and sex **(D)**. Box and whiskers plot. Line represents median, and whiskers minimum-maximum. * Significantly higher (*p* < 0.001) compared to the other group(s).

There was a significant main effect for device (*F* = 12.00, *p* < 0.001), and age (*F* = 4.31, *p* = 0.001) on ADH (Figure 2). On average, ADH was 20% higher for pedometer users (56%) as compared to smartphone application users (36%; 95% CI = 17–23%). Additionally, ADH was higher in 26–35 y (45%) (*p* = 0.008, 95% CI 1–7%), 36–45 y (47%), (*p* < 0.001, 95% CI = 3–9%), 46–55 y (51%, *p* < 0.001, 95% CI = 5–13%), and 56–65 y (*p* = 0.002, 95% CI = 3–12%) compared to 16–25 y (41%). Furthermore, ADH increased significantly between 26–35 y and 46–55 y (*p* = 0.003, 95% CI = 2–9%). There were no significant main effects for sex, BMI or nationality on ADH, and no interaction effects (*p* ≥ 0.21).

### Retention

**Figure 3:**
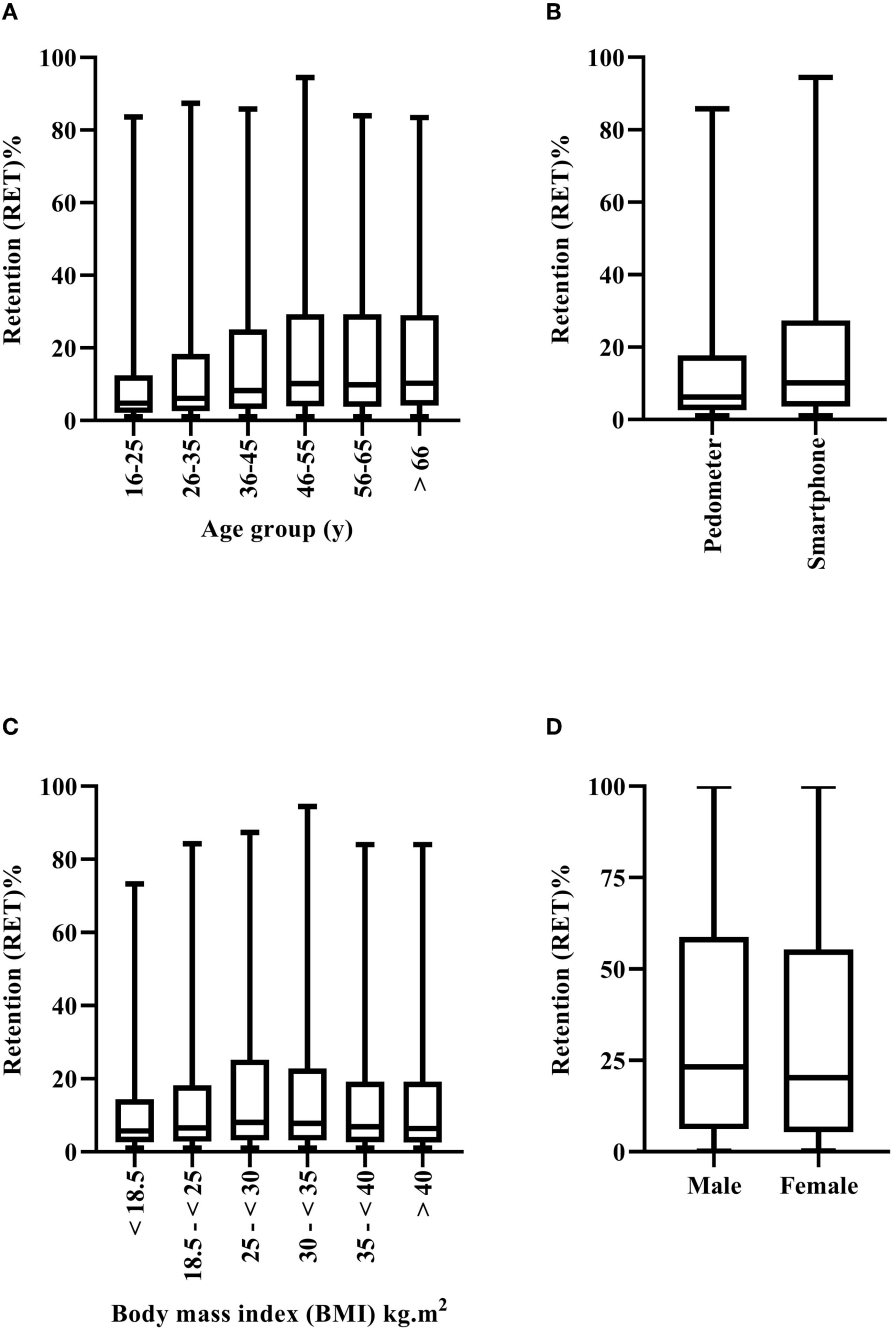
Retention (RET) to the step into health (SIH) program by age group **(A)**, device used **(B)**, body mass index [(BMI), **(C)**] and sex **(D)**. Box and whiskers plot. Line represents median, and whiskers minimum-maximum.

Overall, the average RET to the SIH program was 16% (±20%) (i.e., 463 d). There was a significant difference in RET between Arab's and non-Arab's (*F* = 5.37; *p* = 0.02). On average RET was 17% in Arabs and 16% in non-Arabs (*p* = 0.02; 95% CI = 0.1–1%) see (Figure 3). There was a significant association between RET and sex (*p* < 0.001), device (*p* < 0.001), and age groups 16–25 y (*p* < 0.001), and 26–35 y (*p* < 0.001). The odds of females being in a higher RET category decreased by −0.83 [(95% CI = −0.12 to −0.25), Wald $\chi_{\left(1\right)}^{2}$ = 32.2, *p* < 0.001]. Additionally, the odds of pedometer users being in a higher RET group increased by a factor of 1.63 [(95% CI = 1.53–1.73), Wald $\chi_{\left(1\right)}^{2}$ = 240.0, *p* < 0.001]. Finally, the odds ratio indicated that the odds of being in a higher RET group decreased by a factor of 0.37 and 0.51 for every one unit increase in age in 16–25 y [(95% CI = 0.26–0.53), Wald $\chi_{\left(1\right)}^{2}$ = 28.5, *p* < 0.001], and 26–35 y olds [(95% CI = 0.36–0.73), Wald $\chi_{\left(1\right)}^{2}$ = 13.8, *p* < 0.001, respectively]. There were no signification associations between any other age groups or BMI (*p* ≥ 0.12).

## Discussion

The main findings in the present study were that overall ADH and RET to the SIH program (irrespective of sex, age, device and BMI) was 50% (±30%) and 16% (±20%) (i.e., 463 d), respectively. Additionally, pedometer users, and older age groups had higher ADH to the SIH program. Furthermore, RET (i.e., the number of days a participant stayed in the SIH program) was higher in males, pedometer users and non-Arab's. However, older age groups had lower RET to the program. Furthermore, ADH was greater in the lowest (30–293 d), and highest (1,464–2,928 d) RET groups, indicating that participants were highly motivated in the short-term (i.e., in the early stages of enrollment), and in the longer-term (i.e., committed users), with intermittent use within the middle. These findings are in agreement with previous research showing three distinct groups (i.e., short-term, intermittent, and long-term committed) (16) of wearable tracker users. Additionally, similar ADH values were reported in the present study (i.e., 50% on average) compared to previous longitudinal research showing daily adherence of 20–100% (22) (see Figure 1). However, average RET in the present study (i.e., 16%), was lower than typical values reported across the literature, indicating a higher attrition rate to the SIH program.

In contrast to the hypothesis, ADH was higher for pedometer users. Previous research had suggested that wearing a pedometer on the hip, may have been a barrier to ADH (31, 32). However, offering participants a free pedometer may have provided a sense of responsibility toward the program, and enhanced motivation. Additionally, there are limitations to the SIH smartphone app. Data from the app has to be uploaded on a daily basis, compared to every 20 days using the pedometer, which could have been a factor in the lower ADH for app users. Pedometer use also increased the odds of greater RET, suggesting that those using the pedometer were more likely to be long-term adherers to PA tracking. Smartphone apps designed based on behavior change techniques (i.e., self-monitoring, goal setting, feedback) may be limited in their ability to engage users (33), which could partly explain the findings in the present study. Subsequently, specific smartphone apps designed to encourage more walking and increased adherence to PA are required. These apps would need to de developed taking into account differences in sex, age, socio-economic status, and other factors and behaviors identified in the specific region they are to be used. For example, as aforementioned due to the internationalization within the state of Qatar, specifically Arab's across various MENA regions possessing different values and beliefs in terms of PA. It is important that smartphone apps capture these differences, if they are to be effective in behavior change and long-term adherence to PA. As shown in the present study RET was higher (17%) in non-Arab's. Future research should be conducted to understand the reasons for this.

In agreement with the hypothesis and previous research (11, 34–37), there was higher ADH for older age groups. Conversely, RET was worse for the older age groups. Compared to the younger ages groups (16–25 y, and 26–35 y), as age increased, participants were less likely to have higher RET. This finding suggests that as age increased, the length of time a participant was in the SIH program decreased. However, as ADH was higher, although the time they were retained in the program was worse, their ADH for the shorter time, was better than the younger age groups. Subsequently, age specific interventions need to be developed, to understand why younger age groups are more likely to continue (i.e., higher RET) in the program, but be worse at adhering to it. And conversely, why older age groups are much better at adhering to the program, but tend to lapse quicker (i.e., lower RET). Future research (e.g., employing interviews, focus groups etc.) is required in order to understand the barriers and facilitators, based on age, as a first step to developing targeted public health initiatives or interventions across the state of Qatar.

Finally, in contrast with the hypothesis, there was similar ADH for males and females (Figure 2) to the SIH program. Conversely, RET (i.e., number of days in the program) was higher for males compared to females (Figure 3). Similar findings have been reported across previous studies (11, 35). Lower RET to a community-based PA program such as SIH is unsurprising, given that Arab females are highly inactive, and may face additional barriers to engaging in PA. Indeed, Islamic traditional clothing (i.e., Abaya and Hijab), adopted widely by Arab females in public places, has been considered an additional barrier regarding engagement in PA (38). Female students previously reported they did not like to wear sports clothes underneath their Abayas (39). Additionally, women traditionally need to be accompanied by a male family member when going outdoors (40). Such factors may have made it more difficult for Arab females to continue their engagement with the SIH program. The SIH program's marketing and publicity strategies were predominately focused on “10,000 steps,” “walking outdoors,” “move more, sit less, and be active.” Although walking is a culturally appropriate activity for Arab females. The majority of working Arab women are employed in office-based environments, where they are seated for prolonged periods. Given that lack of time, facilities, or social support are longstanding barriers to PA participation (41, 42), and that Arab women may face additional barriers such as limited access to female only facilities, their care-giving roles and other cultural restrictions (7, 43, 44), the SIH program may have benefited from differentiated and targeted marketing to maintain the RET of Arab women in the workplace (45), or indeed within the home, as opposed to the marketing strategies employed. Future research employing focus groups, interviews etc. in this population are required, in order to understand these factors further and develop appropriate solutions. For example, a culturally appropriate and specifically targeted SIH app for Arab women in Qatar, that considers all the potential barriers and factors (e.g., time facilities, weather, support), which can be utilized at home and work, could be developed to increase RET to PA.

Overall, these findings agree with previous research that has demonstrated high variation, and diverse patterns of ADH and wearing behavior (12) within sexes, age, environment (46, 47), time of day (47, 48) and day of week (47, 48). Follow-up studies are required (e.g., interviews, focus groups), to ascertain the reasons for the differences in ADH and RET reported in this study, and to convert those that lapsed and/or abandoned PA tracking to committed long-term adherers. Long-term ADH (i.e., several months to years) to PA monitoring with wearable technology is possible (22, 48). However, as multiple factors contribute to ADH including forgetting to wear the device, device loss, or changes in motivation to track (12), it is essential to ascertain the specific reasons for each age, sex, community etc. Additionally, as people often lapse or abandon tracking due to their goals not being met (16), it is imperative that researchers, public health advisors and organizations using wearable technology at a population level, communicate and educate the individuals engaging in the research and/or interventions to ensure their needs and expectations are met. Perhaps inviting people to review the data they previously collected, offering support and encouragement could motivate them to resume actively tracking (16).

### Strength and limitations

The SIH program was a longitudinal national community-based self-managed PA initiative employing an objective measure of PA (i.e., smartphone app and pedometer). The objectivity, large sample size, and longitudinal nature (2012–2019) is a strength of this present study. The study is limited, as it did not employ interview or focus groups to understand participants' motivations, knowledge and reasons for ADH, lapsing or abandonment of the program. Participants may have still been using the app or pedometer but not uploading the data to the website. Additionally, although the pedometer utilized (Omron HJ-324U) has been shown to be accurate (when worn at the waist, chest and armband) in measuring step count, irrespective of terrain and walking speed (25). The reliability, error rate and location of the pedometer was not measured in the present study. Furthermore, actual step count is highly varied between and within smartphone apps (49) due to a number of reasons including wearer location (50, 51) and age (52). Such data was not examined in the present study, and therefore findings should be interpreted with caution. Future research, initiatives and interventions will employ such strategies, and follow up with participants in order to better understand the factors and barriers that individuals across the state of Qatar face when engaging in such programs. This will allow evidence based public health strategies to be developed in order to increase PA at a population level.

## Conclusion

Overall, ADH and RET to the SIH program (2012–2019) was 50%, and 16% (463 d), respectively. Device, age and sex affected ADH and RET to the self-managed community based program. Despite ADH being similar between males and females, the dropout rate (i.e., RET) was greater for females. Similarly, older age groups showed higher ADH but also higher attrition. Free pedometer use increased ADH and RET, despite some of the previously suggested barriers to wearing an accelerometer. The factors and barriers associated with ADH and RET in a diverse Arab population, remain to be elucidated. Further research (e.g., focus groups, interviews) is required in order to ascertain the reasons why certain individuals or groups engage and/or dropout of community-based PA programs. This will provide essential information in order to appropriately design, market, and conduct population based interventions and initiatives across the state of Qatar to increase quality of life and reduce morbidity and mortality.

## Data availability statement

All relevant data is contained within the article. The original contributions presented in the study are included in the article. Further inquiries can be directed to the corresponding author.

## Ethics statement

Ethical approval was obtained from Qatar University Institutional Review Board (QU-IRB 1150-EA/19). The patients/participants provided their written informed consent to participate in this study.

## Author contributions

BC performed the statistical analyses, created the figures, and wrote the initial manuscript. LM contributed in planning the analyses and in the revision and approval of the submitted. SS and AA-M contributed in obtaining the data, planning the analyses, and in the revision and approval of the submitted version. All authors contributed to the article and approved the submitted version.

## Funding

Open Access funding provided by the Qatar National Library.

## Conflict of interest

The authors declare that the research was conducted in the absence of any commercial or financial relationships that could be construed as a potential conflict of interest.

## Publisher's note

All claims expressed in this article are solely those of the authors and do not necessarily represent those of their affiliated organizations, or those of the publisher, the editors and the reviewers. Any product that may be evaluated in this article, or claim that may be made by its manufacturer, is not guaranteed or endorsed by the publisher.
